# H2AX phosphorylation screen of cells from radiosensitive cancer patients reveals a novel DNA double-strand break repair cellular phenotype

**DOI:** 10.1038/sj.bjc.6605666

**Published:** 2010-05-11

**Authors:** R S Vasireddy, C N Sprung, N L Cempaka, M Chao, M J McKay

**Affiliations:** 1 Division of Research, Peter MacCallum Cancer Centre, St Andrews Pl., Melbourne, Victoria 3002, Australia; 2 Department of Pathology, University of Melbourne, Melbourne, Victoria 3010, Australia; 3 Department of Biochemistry and Molecular Biology, University of Melbourne, Melbourne, Victoria 3010, Australia; 4 Centre for Women's Health Research, Monash Institute for Medical Research, Monash University, 246 Clayton Road, Clayton, Victoria 3168, Australia; 5 Radiation Oncology Victoria, East Melbourne, Victoria, Australia; 6 Department of Radiation Oncology, The Canberra Hospital, Building 3, Level 1, Yamba Drive, Garran, ACT 2606, Australia; 7 The Australian National University, Canberra, ACT 2600, Australia

**Keywords:** *γ*H2AX, radiosensitivity, ionising radiation

## Abstract

**Background:**

About 1–5% of cancer patients suffer from significant normal tissue reactions as a result of radiotherapy (RT). It is not possible at this time to predict how most patients’ normal tissues will respond to RT. DNA repair dysfunction is implicated in sensitivity to RT particularly in genes that mediate the repair of DNA double-strand breaks (DSBs). Phosphorylation of histone H2AX (phosphorylated molecules are known as *γ*H2AX) occurs rapidly in response to DNA DSBs, and, among its other roles, contributes to repair protein recruitment to these damaged sites. Mammalian cell lines have also been crucial in facilitating the successful cloning of many DNA DSB repair genes; yet, very few mutant cell lines exist for non-syndromic clinical radiosensitivity (RS).

**Methods:**

Here, we survey DNA DSB induction and repair in whole cells from RS patients, as revealed by *γ*H2AX foci assays, as potential predictive markers of clinical radiation response.

**Results:**

With one exception, both DNA focus induction and repair in cell lines from RS patients were comparable with controls. Using *γ*H2AX foci assays, we identified a RS cancer patient cell line with a novel ionising radiation-induced DNA DSB repair defect; these data were confirmed by an independent DNA DSB repair assay.

**Conclusion:**

*γ*H2AX focus measurement has limited scope as a pre-RT predictive assay in lymphoblast cell lines from RT patients; however, the assay can successfully identify novel DNA DSB repair-defective patient cell lines, thus potentially facilitating the discovery of novel constitutional contributions to clinical RS.

Radiotherapy (RT) is one of the main strategies for cancer treatment, and is a prime contributor to cancer patient survival and loco-regional tumour control (Morgan *et al*, 2004). Radiotherapy dose intensities are typically selected to avoid an unacceptable frequency of severe adverse normal tissue reactions and hence, most patients could potentially tolerate RT dose escalation. This is expected to significantly improve loco-regional cancer control and patient survival. Identification of predictive markers of radiosensitivity (RS) may enable individualisation of RT, which till date has not been achieved in clinical practice.

Many assays with the goal of predicting RS in normal tissues before RT have been attempted, with modest success. Assays that have shown promise include colony survival assays (Brock *et al*, 1995; West *et al*, 1998), chromosomal aberration frequency (Neubauer *et al*, 1997, 2002; Distel *et al*, 2006), comet assay (Brammer *et al*, 2001), DNA damage and repair based on pulsed-field gel electrophoresis (PFGE) (Wurm *et al*, 1994; McKay and Kefford, 1995; Zhou *et al*, 1998), micronucleus assay (Nachtrab *et al*, 1998; Sprung *et al*, 2005), telomere length (McIlrath *et al*, 2001; Sprung *et al*, 2008), SNP analysis (Severin *et al*, 2001; Gurska *et al*, 2007; Wilding *et al*, 2007; Alsner *et al*, 2008), DNA end binding complexes (Ismail *et al*, 2004) and transcriptional profiling (Rieger *et al*, 2004; Svensson *et al*, 2006; Sprung *et al*, in preparation). Thus, many potential endpoints exist, most of which have been partially successful in identifying clinical RS. Given the probable diversity in molecular pathogenesis of RS and the results from these cellular biophysical and molecular assays, most assays will probably be useful only for a minority of patients. A combination of selected assays may ultimately be successful.

Cancer patients are exposed to ionising radiation (IR) during RT treatment, which causes several types of cellular damage, of which DNA double-strand break (DSB) damage is the most significant, as DSBs can result in clastogenesis, mutagenesis and cell death by diverse mechanisms, including mitotic catastrophe, deletions and/or mutations (Sprung *et al*, 2002). An early event in DNA DSB repair is the phosphorylation of histone H2AX (*γ*H2AX) at the DSB site, which in less than an hour encompasses a region spanning several megabases (Rogakou *et al*, 1998), forming a light-microscopically visible focus (Rogakou *et al*, 1999). H2AX protein is phosphorylated on the carboxy terminal at serine 139 by DNA-PKcs, ATM or ATR (Rogakou *et al*, 1998; Paull *et al*, 2000; Burma and Chen, 2004). The number of DSBs can be directly determined by the number of foci present in the cell shortly after DNA damage (Sedelnikova *et al*, 2002). Cells either deficient in or with mutations in the large signalling protein kinases that phosphorylate H2AX are typified by radiation sensitivity, DNA repair defects and genomic instability (Shiloh, 2001). Phosphorylation of H2AX at the site of a DSB induces chromatin remodelling, further amplifying the signal response cascade and resulting in downstream events such as cell cycle arrest, checkpoint activation and cell death (Shiloh, 2003; McGowan and Russell, 2004). *γ*H2AX has been found to recruit various repair factors to the site of DNA damage, has an anchoring function and also retains the broken chromosomal ends in close proximity (Bassing and Alt, 2004). Although initial migration of various signalling and repair factors to DSBs occurs very rapidly (Nelms *et al*, 1998), accumulation into visible IR-induced H2AX foci becomes apparent long after DNA damage, with co-localisation of 53bp1, Mdc1, Mre11, Rad50, Nbs1, Rad17 and Brca1 to foci developing afterwards (Paull *et al*, 2000; Stucki and Jackson, 2006; Spycher *et al*, 2008). *γ*H2AX-deficient cell lines can maintain DSB-induced signalling, and *γ*H2AX formation is not necessary for non-homologous end joining and homologous recombination-mediated DNA DSB repair, but these processes have decreased efficiency in such cells (Petersen *et al*, 2001; Bassing *et al*, 2002; Celeste *et al*, 2003).

The half-life of *γ*H2AX foci after DNA damage is approximately 2–7 h in various cell types (MacPhail *et al*, 2003; Bouquet *et al*, 2006). Focus loss may be because of non-repair events that contribute to assay background. Although selection of appropriate controls can potentially overcome this issue, they may have an impact on assay sensitivity. Bouquet *et al* formally documented, as would be logical to expect, that loss of *γ*H2AX foci can be because of dephosphorylation. Recent genetic and biochemical approaches have shown the role of phosphatases in the removal of the *γ*H2AX phosphate group at DSB sites (Chowdhury *et al*, 2005; Keogh *et al*, 2006). Another possibility is *γ*H2AX exchange, which involves replacement of phosphorylated H2AX with an unphosphorylated H2AX, for which there is evidence in yeast and *Drosophila* (Kusch *et al*, 2004; Svetlova *et al*, 2007). It has been proposed that the signal for *γ*H2AX depletion is completion of a critical DNA repair step.

Before this study, *γ*H2AX was approved as a marker of DNA damage and repair and was considered likely to be an improved candidate predictive assay, as it is associated with DSBs in their native and post-irradiation modulated chromatin and is a main lesion following radiation, with foci being easily visualised and quantitated. In addition *γ*H2AX foci are assayed *in situ* and can be observed in a reasonable time frame following IR treatment, which is an important clinical requirement for a predictive assay. As there is a lag time to full completion of DNA DSB repair, as measured by *γ*H2AX foci assays, it is believed that the recovery of non-phosphorylated H2AX must be associated with factors other than just the rejoining of broken DNA, such as proper chromatin organisation (Olive and Banath 2004).

*γ*H2AX foci kinetics have been examined in a number of RS cellular models. Tumour cell lines of lower RS showed slower rates of *γ*H2AX depletion than more RS cell lines (Banath *et al*, 2004; Olive and Banath, 2004; Taneja *et al*, 2004). Likewise, H2AX phosphorylation depletion was found to be slower in cells with higher intrinsic RS (Olive and Banath, 2004). Increased cellular RS in 18 tumour cell lines, as measured by clonogenic survival, correlated with increased residual *γ*H2AX foci (Klokov *et al*, 2006) and other previous DSB repair methods. However, not all studies have found correlations between *γ*H2AX foci and RS. For example, no such correlation was observed in primary fibroblasts from acute RS *vs* control patients (Mahrhofer *et al*, 2006). Moreover, no correlation of *γ*H2AX foci kinetics was observed in peripheral blood mononuclear cells from prostate cancer patients with late effects (Olive *et al*, 2008).

Quantification of *γ*H2AX foci using immunocytochemistry (ICC) is the most sensitive method, with a detection potential of a single focus within the nucleus (Qvarnström *et al*, 2004). Here we use *γ*H2AX ICC methods and our collection of lymphoblast cell lines (LCLs) derived from severe RS patients (both acute- and late- effect cases), using molecular-based assays focusing on DNA breakage and repair in an attempt to identify individuals who show abnormal phenotypes.

## Materials and methods

### Cell culture

Cancer patient lymphocyte cells were isolated from individuals who did and did not have a severe reaction to RT, and LCLs were derived as previously described (Neitzel, 1986; Severin *et al*, 2001; Sprung *et al*, 2005). Radiosensitive LCLs were derived from patients who had been classified as Radiotherapy Oncology Group (RTOG) 3 or 4; LCLs classified as RTOG 0-1 were selected for use as controls. The ataxia telangiectasia (AT)-deficient LCLs were derived in the same manner. The ligase IV human knock-out LCLs were derived from a Nalm6 cell line as described previously (Distel *et al*, 2003; Sprung *et al*, 2008). The clinicopathological patient characteristics are presented in Table 1. Lymphoblast cell lines were grown in RPMI-1640, 10% FBS and 20 *μ*g ml^−1^ of gentamicin in a humidified 5% CO_2_ environment.

### *γ*H2AX foci immunofluorescence

After two washes in phosphate-buffered saline (PBS: 137 mM NaCl, 2.7 mM KCl, 10 mM Na_2_HPO_4_, 2 mM KH_2_PO_4_), LCLs were plated at a concentration of 7 × 10^5^ cells per ml in fresh RPMI. The cell lines were coded, and so the results were obtained without knowledge of the RS status of the patient from whom they were derived. Cells were exposed to 0  Gy, 1, 2 or 4 Gy of gamma radiation from a ^137^Cs source at a dose rate of 0.62 Gymin^−1^ on ice and then placed at 37°C for 1 h. Aliquots of 0.7 × 10^5^ cells were cytospun for 5 min at 500 r.p.m. onto polysine slides (Menzel-Glaser, Braunschweig, Germany). The slides were fixed for 5 min with 4% paraformaldehyde (Sigma-Aldrich, St Louis, MO, USA), rinsed with PBS, and then the cells were permeabilised for 5 min in 0.1% Triton X-100 followed by three 5-min PBS washes. Slides were treated thrice for 10 min in a blocking solution of 1% bovine serum albumin (BSA) (Invitrogen, Molecular Probes, Eugene, OR, USA). Mouse anti-*γ*H2AX antibody (Upstate, Lake Placid, NY, USA) was added (1 : 500 in 1% BSA) and incubated for 2 h in a dark room-temperature-humidified environment. Slides were then exposed to a secondary goat anti-mouse antibody (1 : 500 in PBS) conjugated with Alexa-488 (Molecular Probes) and incubated for 1 h in the dark room-temperature-humidified environment. Slides were rinsed thrice in PBS and stained with 0.2 mg ml^−1^ of 4′,6-diamidino-2-phenylindole (DAPI, Sigma). Using an Olympus microscope with an × 60 oil immersion objective (Olympus IX81, Tokyo, Japan), *γ*H2AX foci images were acquired using a constant exposure time of 500 ms. Eight to ten z-sections with a 0.5-*μ*m step size were deconvoluted to obtain *γ*H2AX foci images for counting using Metamorph (Molecular Probes), by applying morphological filters, Top-hat and H-dome with constant thresholds set at 40 (low) to 1500 (high). Foci count primary data are presented in Supplementary Table S1. Blood collection from patients was approved by the Peter MacCallum Cancer Centre Ethics Committee and informed consent was obtained from all patients.

### PFGE assay

^3^H-thymidine was added to log-phase LCLs and the cells were allowed to grow for an additional 24 h at 37°C. Lymphoblast cell lines were washed twice with PBS solution (1.4 M NaCl, 0.02 M KCl, 0.05 M Na_2_HPO_4_ and 0.02 M NaH_2_PO_4_). Approximately 4 × 10^5^ cells per ml from each cell line were irradiated at 40 Gy on ice, and then transferred to a 37°C incubator for time points of 0, 30, 60, 120 and 240 min. At these time points, aliquots of 2 × 10^5^ cells were mixed in 0.8% low-melting-point (LMP) agarose diluted in PBS, and 50 μl plugs were prepared and stored at 4°C for 20 min until lysis. Lysis was carried out using ice-cold lysis buffer (2% sarkosyl, 1 mg ml^−1^ proteinase K, Promega, Madison, WI, USA, in 400 mM Na_2_EDTA, pH 8) for 1 h at 4°C and then incubated at 50°C for 20 h. Plugs were washed thrice for 30 min in 0.1 M EDTA and equilibrated in running buffer (45 mM Tris base, 45 mM boric acid, 1 mM Na_2_EDTA, pH 8 (TBE)) for 30 min before PFGE. 0.8% gels were prepared and run in 0.5 × TBE gel running buffer. Plugs were sealed with 0.8% LMP agarose and placed in a Gene Navigator (Pharmacia Biotech, Uppsala, Sweden) PFGE unit. Electrophoresis was performed at 6.5 V cm^−1^ at 14°C, with a pulse rate of 5 and 8 s for 3 h each. To view the fraction of DNA released into the gel, the gel was stained using SYBR Gold (Invitrogen, Carlsbad, CA, USA) and imaged using a Molecular Imager FX (Bio-Rad, Hercules, CA, USA). The fraction of DNA released from the gels was cut using SYBR gold stain as a template, and solubilised in concentrated HCl, mixed with a liquid scintillation cocktail (Beckman Coulter, Fullerton, CA, USA) and counted using a tri carb TR2100 scintillation counter (Packard, Meriden, CT, USA).

## Results

**Figure 1:**
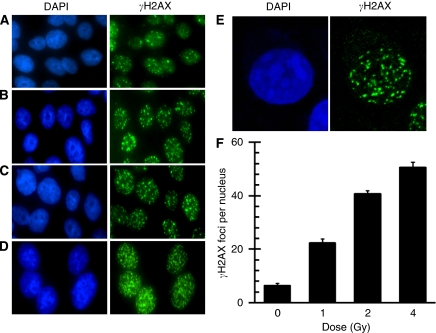
Dose response of *γ*H2AX focus formation in lymphoblast cell lines (LCLs). Immunocytochemistry (ICC ) was used to capture representative *γ*H2AX focus images from asynchronous log-phase non-radiosensitive (RS) control cells (control-5) before (**A**) and 1 h after 1 Gy (**B**), 2 Gy (**C**) and 4 Gy (**D**) of ionising radiation (IR). (**E**) A more detailed view of *γ*H2AX focus distribution, apparently not limited to either euchromatin or heterochromatin in a cell exposed to 2 Gy of IR. Slides were counterstained with DAPI to visualise nuclei (left panels). *γ*H2AX foci were quantified as foci per nucleus for each dose (**F**). Error bars are the standard error of the mean (s.e.m.) of *γ*H2AX foci number per nucleus from three separate experiments, wherein at least 100 nuclei cells were scored per dose.

*γ*H2AX foci kinetics were examined in a LCL (control-5; Table 1) 1 h following treatment with graded IR doses up to 4 Gy. We found a clear and roughly linear dose response, consistent with the data reported by others in this cell type (MacPhail *et al*, 2003, Figure 1). We observed six *γ*H2AX foci per nucleus on an average for this cell line before IR, which is typical for endogenous foci (Vilenchik and Knudson, 2003). Irradiation with 1, 2 and 4 Gy (Figure 1B–D, respectively) resulted in an average of 22, 41 and 50 foci per nucleus, respectively, 1 h after IR. A relative decrease in *γ*H2AX foci number per Gy and an increase in foci overlap was exhibited at 4 Gy. As doses of 1 and 4 Gy tended to be non-linear because of foci overlap, we used 2 Gy for our screening assay, which was consistent with previous reports that showed 2 Gy to be at the upper end of the linear response range for counting *γ*H2AX foci using ICC methods (MacPhail *et al*, 2003).

**Figure 2:**
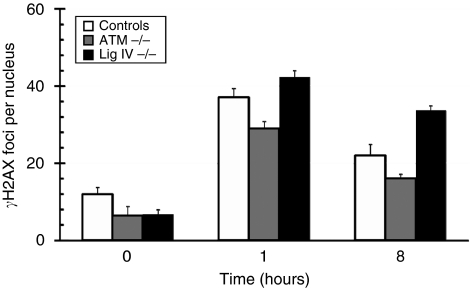
Time-course kinetics of DNA repair-deficient LCLs. *γ*H2AX foci per nucleus are shown for controls (*n*=11; open bars), homozygous ATM mutant (three experimental replicates; grey bars) and ligase IV knock-out (three experimental replicates; black bars) human cell lines. *γ*H2AX foci number was counted in cells before (0 h) and 1 and 8 h after exposure to 2 Gy of IR. Error bars represent s.e.m. of *γ*H2AX foci number per nucleus.

To confirm the reliability of the ICC assay, we tested LCLs with a known cellular RS phenotype, namely, LCLs with a ligase IV homozygous deficiency and also an ATM-deficient cell line. We found that the ligase IV-deficient cell line had a higher number of foci when compared with the controls (*n*=11) at 1 h following IR, with over 30 *γ*H2AX foci per nucleus after 8 h, much higher than the number of foci found in controls, indicating a slow repair rate for these cells (Kuhne *et al*, 2004) (Figure 2). The ATM-deficient cell lines did not have any significant differences in foci induction or depletion as compared with the controls (Figure 2). This is also consistent with previous findings that, although considerably RS, ATM-mutated homozygous cell lines repair bulk DNA DSBs with similar kinetics to control cells, but have an elevated number of residual, perhaps complex, DNA DSBs (Kuhne *et al*, 2004).

**Figure 3:**
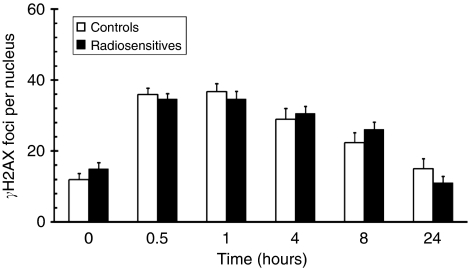
Time-course kinetics of IR-induced *γ*H2AX foci in LCLs derived from clinically RS individuals (*n*=18) *vs* control LCLs (*n*=11). *γ*H2AX focus number was measured in control (open bars) and RS (filled bars) cell lines before (0 h) and at various times after 2 Gy of IR. Error bars represent s.e.m. of foci number per nucleus based on 4–5 fields of approximately 20–25 cells per field. Data have been graphed from 0 to 8 h for all RS and CL cell lines (controls: *n*=11; RS: *n*=18), and at 24 h for a random subset of cell lines (controls: *n*=5 [CL1, CL3, CL4, CL9, CL11] RS: *n*=13 [A1, A2, A3, A4, A6, A7, L3, L5, L6, L7, L9, L11]) (Table 1).

We aimed to determine whether there was a link between clinical RS and the response to radiation as measured by *γ*H2AX ICC. Therefore, we analysed a relatively large cohort of 18 LCLs derived from RT patients who showed severe (RTOG⩾3) adverse normal tissue RT reactions and 11 non-RS control cancer patients. Background foci varied between the cell lines, with a range of 4–23 and a median of about 12 foci per nucleus. Treatment with 2 Gy of IR yielded around 35 foci per nucleus on an average within the first hour (Figure 3). There was a slow decline in visible *γ*H2AX foci, which resulted in about 20–25 foci per nucleus on an average, after 8 h post-IR (Figure 3). The average rate of *γ*H2AX depletion from the first hour to 8 h following IR was 2 foci and 1.2 foci per hour for controls and RSs, respectively. A subset of these samples (*n*=CL-5; RS=13) were analysed at 24 h post-IR and showed a further decrease in foci number, approximately to background levels (Figure 3). Statistical analysis revealed no significant difference between control and RS samples, on average.

**Figure 4:**
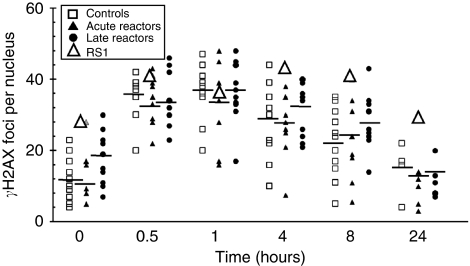
Time-course kinetics of *γ*H2AX focus induction and repair for each LCL following IR. *γ*H2AX focus numbers per nucleus before (0 h) and at various times after 2 Gy are plotted for each control cell line (open squares), acute (filled triangles) and late (filled circles) RS patients. RS1 is designated as an open triangle.

**Figure 5:**
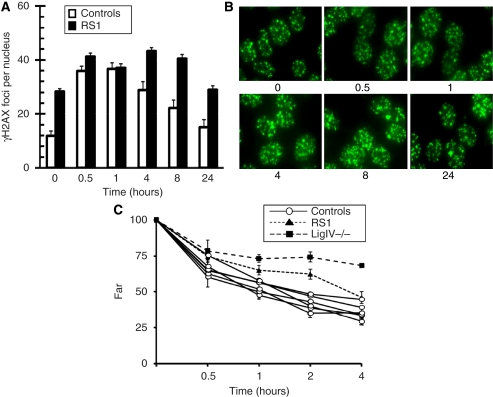
Dynamics of DNA repair in LCLs from an RS individual (RS1) following IR. (**A**) *γ*H2AX focus numbers for unirradiated (0 h) cells and irradiated cells at various time intervals after 2 Gy of IR are shown for control cell lines (open bars) and the RS1 cell line from two separate experiments (filled bars). Error bars represent s.e.m. of foci number per nucleus determined from 11 control cell lines and two replicates of RS1. (**B**) Representative *γ*H2AX ICC focus images are shown for asynchronous log-phase RS1 cells before and at the indicated times (hours) after 2 Gy of IR. (**C**) Pulsed-field gel electrophoresis (PFGE) was carried out on RS1 (filled triangles), and a ligase IV-deficient cell line (filled squares) after various time points (in hours) along with six controls (open circles; CL1, CL2, CL5, CL9, CL10, CL11) following 40 Gy of radiation. Error bars represent the differences in mean from two separate experiments, each determined from two replicates run on separate gels.

Clinical RS can be classified into two main types: acute, wherein the adverse effects are apparent during or soon after treatment, and late, wherein the adverse reactions, such as fibrosis, present an adverse phenotype 6 months or longer following radiation treatment. Therefore, we sub-divided our cell lines into these two categories for further analysis (Figure 4). One cell line, RS1, stood out with a relatively slow rate of *γ*H2AX foci depletion after 4, 8 and 24 h. This cell line was subjected to additional testing for *γ*H2AX foci induction. We confirmed the slow *γ*H2AX foci depletion for this cell line in two independent experiments (Figure 5A). The *γ*H2AX foci levels of the RS1 cell line were also checked at a longer time point (24 h) and found to have 29 foci, much higher than any of the controls (*n*=5), which had an average of 15 foci after 24 h of recovery (Figures 4 and 5). We also observed that RS1 had higher basal levels of *γ*H2AX foci (Figure 5A and B), perhaps reflecting genomic instability in this transformed cell line.

We also wanted to confirm, in an independent DNA DSB repair assay, the impaired DNA DSB repair phenotype in RS1 cells, and examined it using PFGE, which directly detects the repair of high-molecular-weight DNA fragments by quantifying DNA release from the wells of an agarose gel. Consistent with the *γ*H2AX foci results, we also found in the RS1 LCLs that there was more residual DNA fragmentation after various times of recovery, relative to controls, confirming impaired DNA DSB repair in the cell line (Figure 5C).

## Discussion

The RS patients’ whose cells were studied here were from phenotypically normal individuals with unexpected severe RT reactions in their normal tissues; no cases had the stigmata of the known RS syndromes, involving genes such as *ATM*, *NBS* and *BRCA*. A subset of the RS cases and controls had previously been screened for mutations in the *BRCA1* and *BRCA2* cancer predisposition/radiation response genes, with no definite mutations being found (Leong *et al*, 2000).

Our results are consistent with others (Kuhne *et al*, 2004) in that we found a clear difference in IR-induced *γ*H2AX foci depletion in a human cell line for which an important gene in DNA repair, DNA ligase IV, has been knocked out, showing the applicability of these molecular methods to RS cell lines. However, we observed no detectable difference between the control group and the RS group as a whole by following *γ*H2AX foci kinetics after IR. Nevertheless, we did find one cell line from the group of RS patient-derived cell lines that showed similar *γ*H2AX kinetics to the ligase IV knockout cell line, which was confirmed by using a direct DNA damage and repair assay. We believe that the *γ*H2AX assay has the potential to perform better than many other cellular RS predictive assays because it detects a very early event, but is still measurable for a reasonable period of time following DNA damage. Moreover, DSBs can be directly correlated with foci, and these can be directly visualised *in situ*. Besides, we noted less variability in the RS1 cell line using the *γ*H2AX assay compared with the PFGE DNA damage and repair assay. Most investigations of cellular response to radiation normally use doses that are well above the threshold of the mean lethal total body dose of 4 Gy in humans. The *γ*H2AX assay circumvents confounding problems that may be associated with dose.

We contend that the slow rate of depletion of visible *γ*H2AX foci in RS1 relative to controls may be because of the factors that led to the patients’ acute clinical RS. The RS1 patient showed a particularly striking acute reaction with strong erythema after only a cumulative 10 Gy dose of IR. We presume that both the high basal foci levels and the DNA repair defect in RS1 are because of the same defect.

We also observed a large inter-cell line range of *γ*H2AX foci at the basal level, as well as at different times following IR. This is consistent with previous reports using the *γ*H2AX assay (Ismail *et al*, 2007), as well as with other RS assays. Therefore, the *γ*H2AX assay used here may not be able to pick up some cell lines with different types or moderate DNA repair defects because of a relatively high variance; however, it still may be better than other common RS assays. It was clearly competent for detecting basal levels predictive of faulty DNA repair. We determined that the loss of *γ*H2AX foci occurs with an half-life of approximately 6 h in control LCLs, but foci levels at this time for the RS1 cell line were still as high as the control levels at 0.5 h and 1 h following IR, and had a half life for foci loss that was much slower than that of controls over the first 8 h.

A number of authors have shown using different assays of radiation response that Epstein–Barr virus (EBV) transformation may affect RS. However, alterations to RS have not tended to be systematic, nor to correlate with EBV load. We previously noted that (Lovelock *et al*, 2007):

‘EBV-mediated immortalisation of lymphocytes results in alterations to levels of expression of a number of cell genes, including several transcription factors. Epstein-Barr Virus Nuclear Antigen-3 (EBNA-3) also disrupts the G2/M cell cycle checkpoint. As part of the immortalisation process, the normal cellular functions of lymphocytes are likely to be disrupted…..’.

In an attempt to control one variable in the immortalisation process, we confirmed by western blot that all LCLs used were transformed by the same EBNA2A EBV strain (Lovelock *et al*, 2007). For the clonogenic survival end point, LCL survival may cluster more than expected compared with non-transformed but cycling B-cells, which can show a wider spread of response (Maneerat *et al*, in preparation). By using the *γ*H2AX assay as we have shown here, it is unknown whether clustering of data (i.e., just one clear outlier) may have been less if non-transformed cells had been used for the screen. However, the fact that ligase IV control LCLs were a clear outlier suggests that the assay as performed can pick up true RS independent of EBV transformation. Ideally, to resolve these issues, one would like to screen both immortalised and mortal cells from RS patients using assays that characterise a number of radiation-response end points. We did not observe unusually high basal levels of DNA damage in the RS1 cell line using the PFGE DNA damage and repair assay as we did in the *γ*H2AX assay. This may be because the majority of variations in basal level breaks observed are due to the shearing caused during DNA processing, and the true initial DNA DSB levels are probably too low to be detected using the PFGE assay that uses such high IR doses. However, RS1 cells had an obvious lag in repair in the PFGE DNA damage and repair assay, which, even after 4 h, was still relatively high compared with the average of the controls.

The defect in DNA repair in RS1 may be further explored by immunofluorescence co-localisation of other repair factors known to form foci at sites of DNA damage. In addition, expression analysis and the use of DNA repair protein functional studies will be pursued.

In conclusion, a fraction of patients who are predisposed to adverse reactions could be detected using ICC assays that detect *γ*H2AX foci. This application, in combination with other predictive assessments, may eventually facilitate the tailoring of RT regimes to individuals, which could result in lesser side effects of RT and better tumour control.

## Figures and Tables

**Table 1:** Patient characteristics

	**Cancer type**	**Cancer site**	**RTOG**	**Dose received**	**Radiosensitivity**	**Sex**	**Age**
*Controls*							
1	Adenocarcinoma	Prostate	0–1	64/32/5	NA	M	70
2	Adenocarcinoma	Prostate	0–1	64/32/5	NA	M	62
3	IDC	Breast	0–1	50/25/5	NA	F	60
4	IDC	Breast	0–1	46/23/5	NA	F	70
5	Adenocarcinoma	Prostate	0–1	66/33/5	NA	M	82
6	None	Nil	0–1	NA	NA	M	39
7	Adenocarcinoma	Breast	0–1	NA	NA	F	53
8	IDC	Breast	0–1	50/25/5	NA	F	71
9	IDC	Breast	0–1	46/23/5	NA	F	47
10	Adenocarcinoma	Breast	0–1	46/23/5	NA	F	63
11	IDC	Breast	0–1	50/25/5	NA	F	52
							
*Acute reactors*							
1	IDC	Breast	3	50/25/5	Erythema, oedema	F	59
2	SCC	Lung	3	36/12/5	Severe dyspnoea	M	75
3	IDC	Breast	3	18/9/5	Wet desquamation	F	44
4	SCC	Tonsillar	3	50/25/5	Mucositis	M	64
5	SCC	Cervical	3	54/30/5	Sigmoid obstruction	F	72
6	Melanoma	Skin	3	50/25/5	Desquamation	F	54
7	Seminoma	Testicle	3	16.5/11/4	Severe nausea, diarrhoea	M	31
							
*Late reactors*							
1	Adenocarcinoma	Prostate	3	60/30/5	Rectal bleeding	M	61
2	IDC	Breast	3	46/23/5	Fibrosis	F	63
3	Adenocarcinoma	Prostate	3	64/32/5	Rectal bleeding, haematuria	M	63
4	Adenocarcinoma	Prostate	3	64/32/5	Rectal bleeding	M	71
5	Adenocarcinoma	Prostate	3	66/33/5	Rectal bleeding	M	73
6	Adenocarcinoma	Prostate	3	64/32/5	Haematuria	M	67
7	MC	Breast	3	46/23/5	Fibrosis	F	51
8	SCC	Cervical	3	45/25/5	Sigmoid and ureter stricture	F	40
9	LC	Breast	3	50/25/5	Fibrosis, telangiectasia	F	73
10	Adenocarcinoma	Prostate	3	64/32/5	Rectal bleeding	M	78
11	LC	Lung	3	40/20/5	Pneumonitis, dyspnoea	F	69

Abbreviations: F=female; IDC=infiltrating ductal carcinoma; LC=lobular carcinoma; M=male; MC=medullary carcinoma; NA=not applicable; RTOG=Radiotherapy Oncology Group; SCC=squamous cell carcinoma.
