# Supplementary material for: H2AX phosphorylation screen of cells from radiosensitive cancer patients reveals a novel DNA double-strand break repair cellular phenotype
Source: Br J Cancer. 2010 May 11;102(10):1511–8. doi: 10.1038/sj.bjc.6605666 (PMC2869166; doi:10.1038/sj.bjc.6605666)
Supplement: Supplementary Table S1 [file 6605666x1.doc]

**Table S1**: γH2AX foci number in LCLs derived from control and RS individuals

| Cell line |  | Foci number/Nucleus | | | | |
| --- | --- | --- | --- | --- | --- | --- |
|  | 0 Gy | 2 Gy; Time (Hour) | | | | |
|  |  | 0.5 | 1 | 4 | 8 | 24 |
| CL1 | 23 | 39 | 36 | 30 | 28 | 17 |
| CL2 | 12 | 37 | 38 | 22 | 15 |  |
| CL3 | 14 | 30 | 26 | 32 | 20 | 16 |
| CL4 | 5 | 35 | 41 | 16 | 11 | 4 |
| CL5 | 10 | 37 | 41 | 35 | 35 |  |
| CL6 | 20 | 42 | 47 | 44 | 34 |  |
| CL7 | 17 | 39 | 35 | 35 | 31 |  |
| CL8 | 9 | 20 | 20 | 10 | 5 |  |
| CL9 | 7 | 42 | 44 | 39 | 25 | 16 |
| CL10 | 10 | 38 | 36 | 23 | 17 |  |
| CL11 | 4 | 35 | 39 | 32 | 24 | 22 |
| *Avg* | *11.9* | *35.8* | *36.66* | *28.99* | *22.27* | *15* |
| A1 | 30 | 42 | 31 | 48 | 44 | 29 |
| A2 | 5 | 22 | 16 | 7.5 | 5.5 | 2.5 |
| A3 | 7 | 27 | 34 | 34 | 24 | 8 |
| A4 | 5 | 33 | 17 | 26 | 34 | 12 |
| A5 | 16 | 39 | 39 | 38 | 31 |  |
| A6 | 8 | 38 | 45 | 25 | 18 | 3 |
| A7 | 8 | 29 | 34 | 28 | 31 | 12 |
| *Avg* | *11.2* | *32.5* | *32.3* | *29.1* | *24.7* | *10.6* |
| L1 | 23 | 46 | 48 | 36 | 33 |  |
| L2 | 19 | 32 | 31 | 26 | 23 | 13 |
| L3 | 14 | 30 | 17 | 35 | 25 | 11 |
| L4 | 10 | 30 | 35 | 21 | 24 |  |
| L5 | 15 | 44 | 37 | 22 | 14 | 7 |
| L6 | 30 | 23 | 33 | 35 | 26 | 20 |
| L7 | 9.3 | 43 | 39 | 30 | 19 | 10 |
| L8 | 22 | 42 | 39 | 39 | 34 |  |
| L9 | 19 | 34 | 44 | 40 | 31 | 8 |
| L10 | 11 | 32 | 33 | 24 | 26 |  |
| L11 | 17 | 35 | 48 | 35 | 24 | 14 |
| *Avg* | *16.6* | *33.8* | *34.6* | *31.5* | *26.4* | *12.1* |
